# Seston Fatty Acid Responses to Physicochemical Changes in Subalpine Lake Lunz, Austria

**DOI:** 10.1029/2017WR020959

**Published:** 2018-10-29

**Authors:** S. Rasconi, R. Ptacnik, M. J. Kainz

**Affiliations:** ^1^ WasserCluster Lunz‐Inter‐University Center for Aquatic Ecosystem Research Lunz am See Austria

**Keywords:** mountain lakes, seston, nutritional quality, temperature

## Abstract

Rapid increase in lake temperature can cause a shift toward the dominance of warm temperature tolerant species, including Cyanobacteria that are deficient in polyunsaturated fatty acids (PUFA) supporting consumer growth and reproduction. To increase our understanding of how changes in physicochemical lake parameters affect phytoplankton composition and the provision of dietary quality to consumers in subalpine oligotrophic lakes, we conducted a multiannual study (2013–2015) in the 34‐m‐deep Lake Lunz and investigated interannual changes in (a) water temperature, transparency, and lake inflow; (b) seston (<30‐μm particle size class) biomass and taxonomy; and (c) seston nutritional quality, assessed by its PUFA composition. The phytoplankton taxonomic composition within this seston size class varied mostly by changes in physical parameters (temperature, conductivity, lake transparency, and days of full ice cover). The dietary quality of seston varied mostly with lake physical parameters and, to a lesser extent, with phytoplankton taxonomic composition, suggesting that the nutritional quality at the base of the food web in Lake Lunz is likely to respond directly to changes in lake physical parameters. This multiannual data set, combining monthly values for physicochemical variables, grazable phytoplankton composition, and fatty acids in seston, provides nutritional information of how annual weather changes may induce changes at the base of the food web in this and perhaps also other oligotrophic subalpine lakes.

## Key Points

- We investigated how changes in physicochemical lake parameters affect seston composition and dietary quality in the subalpine Lake Lunz
- Seston taxonomy varied mostly with conductivity and lake transparency as well as lake inflow and days of ice cover
- The dietary quality of seston was mainly related to lake physical parameters and the taxonomic composition of phytoplankton

## 1 Introduction

The European Alps consist of numerous alpine and subalpine catchments that drain water and nutrients to streams and lakes, providing important habitats for a large number of species throughout all trophic levels (EEA Report, 2009). Mountain lakes are subject to extreme weather conditions and abrupt environmental gradients, such as rapidly changing precipitation, temperature, and wind conditions (Sánchez‐Hernández & Amundsen, 2015). In recent decades, these geographically vulnerable ecosystems were and still are exposed to environmental degradation, driven mainly by climatic change and deforestation (Beniston, 2003), as recorded in many alpine lakes (Bernstein et al., 2008). Rapid changes in physicochemical characteristics may affect the phenology and taxonomy of planktonic populations (Beniston, 2003) and consequently the nutritional value and overall biological interactions within the food web.

For subalpine lakes in the European Alps, the most evident changes include recent increase in surface water temperature and decrease in the duration of ice cover (Dokulil et al., 2010; Kainz et al., 2017) with later freezing and earlier ice breakup (Magnuson et al., 2000). In addition, oligotrophic conditions typical of mountain lakes may be also concerned by altered and/or increased precipitation events. Higher precipitation is expected to increase lake water inflow from catchment runoff followed by higher terrestrial drainage and consequently higher supply of dissolved organic matter and nutrients to lakes (Hongve et al., 2004; Jeppesen et al., 2009). Due to low nutrients and productivity, the seasonal succession of phytoplankton in mountain lakes is strongly dependent on the precipitation regime for nutrient availability and on ice cover for light availability affecting primary production (Christoffersen et al., 2008).

Biological communities in mountain areas are especially sensitive to impacts of changing weather conditions (Pauli, 2016), and altered temperature regimes can directly affect planktonic populations entailing a shift in their taxonomic composition (Eggers et al., 2012; Heino et al., 2009; Rasconi et al., 2017). Warmer temperature can favor fast growing algal species such as Cyanobacteria and small Chlorophyceae (Rasconi et al., 2015). Cyanobacteria are known to be deficient in essential dietary compounds, such as sterols and polyunsaturated fatty acids (PUFA; Taipale et al., 2013) that are required for zooplankton growth and reproduction (Martin‐Creuzburg et al., 2008, 2012), and some may even become toxic (Jöhnk et al., 2008).

Enhanced nutrient supply to lakes affects primary production either directly by favoring higher phytoplankton biomass (De Senerpont Domis et al., 2013) or, indirectly, by limiting phytoplankton biomass due to higher dissolved organic matter supply that lowers photosynthetically active radiation (Jennings et al., 2012). However, the phytoplankton community composition in oligotrophic lakes is generally more stable and predictable compared to meso‐eutrophic lakes (e.g., Ptacnik, Lepistö, et al., 2008; Ptacnik, Solimini, et al., 2008). As increased concentrations of total phosphorous and nitrogen are major drivers for harmful and toxic algae blooms (Paerl et al., 2016; Paerl & Huisman, 2008), oligotrophic lakes are considered less susceptible to such bloom events. Indeed, alpine lakes are expected to respond differently than lowland lakes (e.g., to increases in nutrient supply and water temperature), and Cyanobacteria blooms are not recurrent; however, these sensitive ecosystems are also vulnerable to changing climatic conditions that can favor growth of algae atypical in alpine lakes and entail taxonomic shifts. Picocyanobacteria are common in oligotrophic lakes and proliferate at low light availability (Callieri & Stockner, 2000). Although nontoxic and more easily ingested by filter feeders than filamentous species, picocyanobacteria are considered as poor quality food that is limited in PUFA (Martin‐Creuzburg & von Elert, 2009). Green algae, however, are of variable nutritional quality for consumers, and even the same species can represent different dietary quality depending on their growth and general conditions (Ahlgren et al., 1990). By contrast, algae more adapted to cold temperature, such as Bacillariophyceae and Cryptophyceae, are rich in long‐chain omega‐3 PUFA (Taipale et al., 2013) and of high food quality. As demonstrated in growth experiments, consumers feeding on high quality algal diet (i.e., PUFA‐rich *Cryptomonas*) resulted in significantly higher somatic growth and clutch size (Masclaux et al., 2009). Moreover, phytoplankton edibility and nutritional quality affect the efficiency at which dietary energy is transferred to herbivores (Dickman et al., 2008) with direct effects on their fitness and reproduction (e.g., Bec et al., 2003; Brett et al., 2009; Koussoroplis et al., 2013, 2014). Thus, shifts in the phytoplankton community composition can affect trophic diet transfer within food webs with nutritional implications for consumers.

Despite studies reporting on mostly negative effects of climate change on lake food webs (Winder & Schindler, 2004), including increasing dominance of Cyanobacteria and nutritionally poor algae in warmer waters (Rasconi et al., 2017), little is known about how physicochemical changes (e.g., temperature, ice‐cover, and transparency) affect seasonal and interannual dietary quality at the base of the food web in mountain lakes. Such phytoplankton communities include mainly species adapted to oligotrophic conditions at relatively low temperatures, such as Cryptophyceae, Bacillariophyceae, Dinophyceae, and Chrysophyceae that are generally considered as high‐quality diet (Taipale et al., 2013). Understanding how stenothermic species of high nutritional quality, such as Cryptophyceae, Chrysophyceae, and Bacillariophyceae that are abundant in Lake Lunz (Malicky, 1985), change with increasing temperature and nutrient concentrations has important trophic implications on the dietary supply of PUFA, thus nutritional quality to consumers at higher trophic levels.

To increase our understanding of the potential for environmental change to alter dietary quality at the base of the food web in oligotrophic mountain lakes, we conducted a 3‐year (2013–2015) study in subalpine Lake Lunz, Austria, and investigated the following: (a) interannual changes in water temperature, transparency, and lake seston inflow; (b) seasonal dynamics of seston (<30 μm as the most likely particle size ingested by planktonic filter‐feeding cladocera; Burns, 1968) biomass and taxonomy; and (c) seston nutritional quality, assessed by fatty acids (FA). Lake Lunz (34‐m maximum depth) was chosen for this study because it is an oligotrophic (mean phosphorous concentration of 4.63 ± 1.67 μg/L), subalpine lake (608 m a.s.l.) with very little human disturbance (McMeans et al., 2015). The planktonic food web of Lake Lunz has been regularly monitored since 2009 during the ice‐free period, and rapid increases in epilimnetic temperature and decreasing duration of the ice‐cover were detected during recent years (Kainz et al., 2017). This study provides thus an opportunity for a multiannual survey of an oligotrophic mountain lake and serves as a *baseline* for other oligotrophic subalpine lakes that also undergo increasingly extreme seasonal weather changes (e.g., Magnuson et al., 2000). In an effort to assess short‐term changes in seston nutritional quality for consumers and planktonic herbivores at higher trophic levels, this study evaluated a multiannual data set combining physicochemical variables, seston composition, and FA of lake seston.

We hypothesize that changes in lake temperature, ice cover (Kainz et al., 2017) and precipitation coincide with changes in (a) lake transparency, seston inflow, and nutrients supply and (b) grazable phytoplankton community composition. Species better adapted to changing conditions and higher temperature, for example, fast growing *r‐*strategists such as Cyanobacteria or small Chlorophyceae, will be favored, and we thus hypothesize a consequent shift toward lower nutritional quality for consumers.

## 2 Materials and Methods

### 2.1 Site Study Description

Lake Lunz (68 ha; 47°51′10″N, 15°3′10″E, 34‐m maximum depth) is a prealpine (located in the Lower Austrian Alps) and subalpine (situated below the timberline) oligotrophic lake (mean phosphorous concentration of 4.63 ± 1.67 μg/L) with very little human disturbance (McMeans et al., 2015). Precipitation is recorded on a daily basis at a weather station located on the lakeshore, and the duration of full ice‐cover is recorded annually. During the winter, the lake is generally ice covered, although the duration of full lake ice cover decreased significantly since 1921 (corresponding to 0.36 days less ice‐cover per year in the period 1921–2015) and with ice‐free winters in 2006 and 2013 (Kainz et al., 2017).

### 2.2 Sampling

Lake sampling was conducted monthly during the ice‐free period over three consecutive years (2013–2015). The lake was not accessible during January of all years, from February to May 2013 and from February to April 2015. All lake samples were collected from an anchored platform situated above the deepest point of the lake (34 m). Physicochemical parameters (i.e., water temperature, oxygen, and conductivity) were measured daily throughout the ice‐free period using a YSI multisonde (Yellow Springs Instruments 6920V2‐2‐O, Yellow Springs, OH) at each meter from 0‐ to 30‐m water depth. Water transparency was assessed using a Secchi disk. Water samples were collected from discrete depths: the epilimnion (0–5 m), metalimnion (5–15 m), and hypolimnion (25 m) during thermal lake stratification using a Ruttner water sampler. When the lake was fully mixed (i.e., November and December of every year), water samples were collected at ~5‐, ~10‐, and ~25‐m depth. Seston was collected by screening particles through a 30‐μm mesh on the boat and was subsequently retained in containers for taxonomic analysis or on filters (GF/C Whatman™ filters; 1.2‐μm pore size) for FA analysis; that is, seston <30 μm was retained as this size fraction includes the most likely particle size ingested by filter‐feeding cladocerans (Burns, 1968) and planktonic herbivores (Brooks and Dodson, 1965). All samples were filtered and processed for chemical analysis the same day. In the laboratory, samples for NO_2_‐N, NO_3_‐N, and NH_4_‐N were again filtered (GF/F Whatman™ filters; 0.7‐μm pore size) and analyzed using a continuous flow analyzer (FlowSys, Systea); total phosphorus (TP) was quantified after persulfate digestion (Wetzel & Likens, 1991), and soluble reactive phosphorus (SRP) was quantified after filtration of lake water on acid‐washed filters (Whatman™ GF/F). TP and SRP were subsequently analyzed following a molybdate reaction (Wetzel & Likens, 1991) at 880‐nm wavelength using a UV/Visible spectrophotometer (UV‐1700). Dissolved organic carbon (<0.2 μm) was analyzed using a total organic carbon analyzer (Sievers 900, GE). Seston inflow was determined by filtering water samples (1 L triplicates) from the main stream entering Lake Lunz on precombusted and preweighted filters (GF/F Whatman™, 0.7‐μm pore size, 25‐mm diameter). The filters were subsequently dried for 48 hr at 50 °C and weighed again to quantify dry weight of the particle load to the lake.

### 2.3 Phytoplankton Taxonomic Composition

From each sample in the euphotic zone (epilimnion and metalimnion), unscreened water (150 mL) was fixed with Lugol, and a subsample (50 mL) was settled following the Utermöhl method (Utermöhl, 1958). Each sample was counted on an inverted microscope (Leica DMI 3000 B) at two different magnification levels (40× and 20×), and at least 400 cells were identified to the genus level. Due to the long‐term storage of the samples, it is possible that some species were underestimated. Phytoplankton biovolumes were assigned using log10 transformed and standardized reference data (Kremer et al., 2014). For the taxonomic composition of seston, only the species with dimensions of <30 μm (i.e., the most grazable phytoplankton size) were considered. Colonial Chrysophyceae and Bacillariophyceae of >30 μm in size may be abundant during the summer in Lake Lunz but were excluded from this analysis. The complete list of genera retained for the study is provided in supporting information S1.

### 2.4 Fatty Acids Analysis

For the determination of the FA composition, seston from epilimnion and metalimnion lake water (3 L triplicates) was filtered (<30 μm) and retained on precombusted and preweighted filters (GF/C Whatman™ filters; 1.2‐μm pore size, 47‐mm diameter), cryogenically frozen (−80 °C), and subsequently freeze‐dried for 48 hr. Lipids and their FA were extracted, derivatized, and analyzed as described in Heissenberger et al. (2010) using a gas chromatograph (Thermo Scientific TRACE GC Ultra) equipped with a flame ionization detector and separated using a Supelco™ SP‐2560 column (100 m, 25 mm i.d., 0.2‐μm film thickness). Excalibur 1.4™ (Thermo Electron Corporation) was used for calculation and, if necessary, manual resetting of the chromatograms. Fatty acid concentrations were calculated using calibration curves based on known standard concentrations (Supelco™ 37 FAME Mix). In this study, we focused on PUFA that are physiologically required by consumers (LIN = linoleic acid, ALA = α‐linolenic acid, EPA = eicosapentaenoic acid, DHA = docosahexaenoic acid) and hence considered a fundamental dietary source supplied by phytoplankton.

### 2.5 Statistical Analysis

Data were analyzed using R (http://www.r-project.org). We used analysis of variance (ANOVA) and Tukey's HSD test to check for significant differences in water transparency, conductivity, and precipitation among the study years and for differences in temperature, oxygen, TP, and NO_2_ concentrations among the investigated depths and among the study years, as well as for differences among the study years in seston FA composition (SAFA = saturated fatty acids, MUFA = monounsaturated fatty acids, PUFA = polyunsaturated fatty acids, HUFA = highly unsaturated fatty acids; i.e., PUFA with ≥20 carbons and more than two double bonds; BAFA = bacterial fatty acids; i.e., odd‐chain carbons and their iso‐ and anteiso‐homologues; and TerrFA = terrestrial fatty acids; i.e., C22:0 and longer chains).

To explore the cause‐effect relationships for seston taxonomic composition over the investigated time period, we used multifactor ANOVA among the biovolume of the identified phytoplankton classes. Interannual variations were explored using *year* as a factor and *season* (based on lake stratification defined by mixing depth) as a cofactor to exclude general seasonal variation in the analysis and highlight significant differences among the years. The following environmental parameters were tested: temperature, conductivity, SRP, NO_2_, dissolved organic carbon, seston at the lake inflow, transparency (Secchi depth), mixing depth, precipitation, and ice cover (monthly average and number of days per year). All data have been log transformed to fulfill assumptions of parametric tests.

Principal components analysis (PCA; R package “vegan”; Oksanen et al., 2007) was used to investigate the repartition on the FA composition during the 3 years of the study and the effect of phytoplankton taxonomic composition. Based on the ANOVA results, significant parameters were used to evaluate the effects of lake physicochemical parameters on the seston composition assessed by phytoplankton taxonomy and on seston PUFA composition using variation partitioning (varpart function in vegan). All phytoplankton and FA data were analyzed using the average values in the euphotic zone (epilimnion and metalimnion) and using the relative frequency values expressed as percentage. The statistically significant value was set at *p* < 0.05.

## 3 Results

### 3.1 Physicochemical Parameters

Lake Lunz was typically ice‐covered for several months; however, occasional winter sampling (twice in early December and once in late February 2014) was possible due to the lack of ice cover during the investigated period. For logistical reasons, more frequent winter sampling was not possible, for example, in 2013 or 2014 with no or only 4 days of lake ice cover, respectively, but 37 days of full ice cover in 2015 (ANOVA, *df* = 1, *F* value = 4.7, *p* < 0.05; Figure S1). Precipitation (Figure S2a) was lowest during 2015 and with less monthly variation compared to the other years of the study. Precipitation was more intense from March to September (average 170.4 ± 112.8, 207.8 ± 143.2, and 108.7 ± 38.9 mm in 2013, 2014, and 2015, respectively), while winter months were dryer (average precipitation from October to February was 112.84 ± 59.4, 143.2 ± 55.5, and 38.9 ± 25 mm in 2013, 2014, and 2015, respectively).

**Figure 1:**
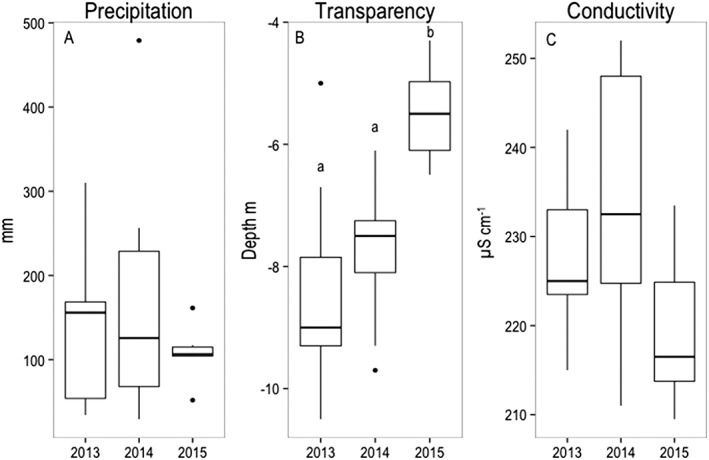
Interannual (2013–2015) variability of (a) precipitation; (b) water transparency assessed by Secchi disk; and (c) conductivity in the epilimnion. The box borders indicate the lower and upper quartiles, the centerline is the mean, and the whiskers extending out from the box indicate the maximum and minimum points outside the upper and lower quartiles. Black dots are outliers. Different letters account for statistical significant differences (ANOVA with Tukey's HSD test *p* < 0.05).

**Figure 2:**
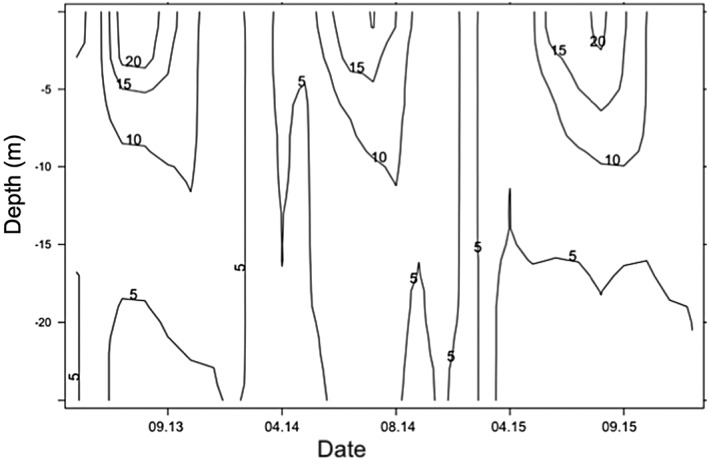
Vertical temperature profile of Lake Lunz during the study years (*x* axis: month.year).

Water transparency (Secchi depth; Figure S2b) decreased during the study years, with the deepest depth recorded in July 2013 (−10.5 m) and the lowest in October 2015 (−4.3 m); in 2015, the Secchi depth was significantly lower compared to 2014 and 2013 (ANOVA, *df* = 2, *F* value = 8.81, *p* < 0.01, Figure 2a). Water transparency was negatively correlated to conductivity (*R* ^2^ = 0.18, *p* < 0.05). Conductivity (Figure S3a) was lower in 2015 (yearly average 219.4 ± 9.1 μS/cm) compared to 2014 (228.8 ± 12.5 μS/cm) and 2013 (227.8 ± 8.8 μS/cm), although not significantly (ANOVA, *df* = 2, *F* value = 1.16, *p* > 0.05; Figure 1c). As for conductivity, seston at the lake inflow had the highest variability in 2014 (9.91 ± 18.4 μg/L), and occasionally high contribution of particulate organic matter from the inflow corresponded to high precipitation peak and low Secchi depth (Figure S2c).

**Figure 3:**
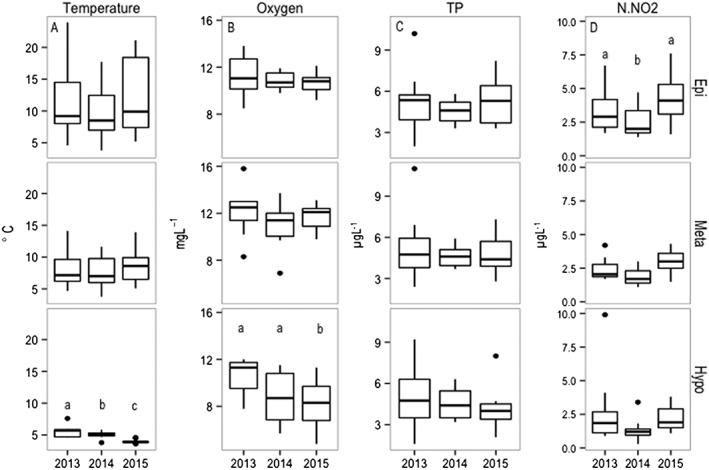
(a) Temperature (°C); (b) dissolved oxygen (mg·g·L^−1^); (c) total phosphorus (TP; μg·g·L^−1^); and (d) nitrogen nitrite (NO_2_‐N) (μg·g·L^−1^) at the three sampling depths (epi = epilimnion, meta = metalimnion, hypo = hypolimnion) during the three study years. The box borders indicate the lower and upper quartiles, the centerline is the mean, and the whiskers extending out from the box indicate the maximum and minimum points outside the upper and lower quartiles. Black dots are outliers. Different letters account for statistical significant difference (ANOVA with Tukey's HSD test *p* < 0.05).

Water column temperatures of Lake Lunz ranged from 3.6 °C in October 2015 to a maximum of 23.9 °C in August 2013 (Figure 2). The time of warmest temperatures and the most stratified water column occurred from June to August with temperatures and water column stratification declining as of September. Surface water temperature (upper 5 m) during this study period was not significantly different among years (ANOVA, *p* > 0.05; Figure 2a), and similarly, the thermocline temperature (metalimnion) and the approximate mixing depth did not also significantly differ (ANOVA, *p* > 0.05). Significantly lower temperatures were recorded in the hypolimnion, where temperatures decreased from 8.6 °C ± 2.8 in 2013 to 4.0 ± 0.3 in 2015 with significantly colder temperatures from 2013 to 2015 (ANOVA, *p* = 0.01 in 2013, *p* = 0.002 in 2014, and *p* < 0.001 in 2015; Figure 3a).

Dissolved oxygen concentrations ranged from 4.8 to 15.8 mg/L, with highest values in the epilimnion during summer and the lowest in the hypolimnion from September to November (Figure S3b). Similar to the observed lake temperatures, oxygen concentrations were not significantly different (*p* > 0.05) in the epilimnion or metalimnion but significantly lower in the hypolimnion (*p* < 0.001; Figure S3b). Oxygen concentrations decreased significantly with increasing temperature in the epilimnion (*R* ^2^ = 0.22, *p* < 0.01), while the predictive power of this correlation decreased in the metalimnion (*R* ^2^ = 0.15, *p* = 0.05) and was not significant in the hypolimnion (*R* ^2^ < 0.1, *p* > 0.05).

Dissolved organic carbon concentrations (1.3 to 9.2 μg/L in the epilimnion during August 2013 and metalimnion in October 2014, respectively) remained rather stable during 2013 and 2014 at the three investigated depths (Figure S3c). Significantly higher dissolved organic carbon concentrations were measured in July (5.6 μg/ml) and October 2014 (8.7 μg/ml; ANOVA, *p* < 0.01).

The highest TP concentration (11 μg/L) was recorded in the epilimnion in October 2013 and the lowest (0.5 μg/L) in the hypolimnion in September 2014 (Figure S3d). Total P concentrations were not significantly different (ANOVA, *p* > 0.05) among the different lake depths and across the three study years (Figure 3c). In the epilimnion, the SRP ranged from a maximum (5.3 μg/L) in July 2013 to very low concentrations (<0.1 μg/L) in November 2014 (Figure S3e).

Nitrite (NO_2_‐N) concentrations (0.3–9.9 μg/L) were generally higher in the epilimnion (Figure S3f) during the summer period and significantly lower in the hypolimnion compared to the upper layers during the last two study years (Figure 3d; ANOVA, *p* < 0.01). Concentrations increased during the study period and were significantly higher in 2013 compared to 2014 (ANOVA, *p* < 0.05) and in 2014 compared to 2015 (ANOVA, *p* < 0.01). Nitrate (NO_3_‐N) concentrations (643–1,142 μg/L) were higher in the hypolimnion (Figure S1 g) with lower values during the summer period (June–July). Ammonium (NH_4_‐N) concentrations (1.7–73 μg/L) did not significantly differ throughout the study period (Figure S3 h).

### 3.2 Seston Taxonomic Composition

**Figure 4:**
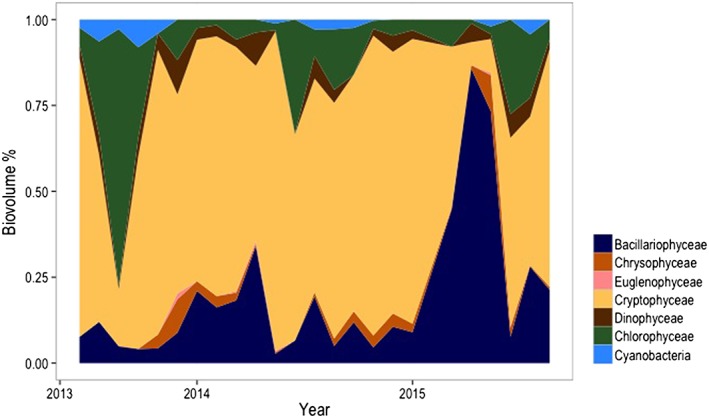
Phytoplankton biovolume relative repartition (%) of the grazable fraction (i.e., <30 μm; the complete list of the included species is provided as supporting information) among the detected taxa during the 3 years of the study.

Cryptophyceae had the highest biovolume among algal groups (Figure 4) present in all samples throughout the study period, notably from September to February, when they were the dominant group representing up to 80% of the total grazable phytoplankton biovolume. The most ubiquitous species were Rhodomonas lacustris and Rhodomonas *nanoplanktonica*, present in 81% and 75% of the samples, respectively. The biovolume of Cryptophyceae significantly changed during the study years (*df* = 2, *F* value = 6.54, *p* < 0.01) and was highest during 2014. Most important factors in determining the Cryptophyceae biovolume were SRP (*df* = 5, *F* value = 8.86, *p* < 0.01) and seston inflow (*df* = 5, *F* value = 8.86, *p* < 0.01; Table 1).

**Table 1:** Analysis of Variance Between Environmental Parameters and Biovolume of Phytoplankton Taxonomic Groups

Tested variable		Year	Temp	Cond	SRP	NO_2_	DOC	Seston inflow	Secchi	Precipi‐tation	Mixing depth	Ice cover
Bacillariophyceae	*df*	1			5			5			2	1
	*F* value	11.08			7.33			8.88			9.72	19.28
	*p*	0.001	ns	ns	0.01	ns	ns	0.01	ns		0.001	<0.001
Chrysophyceae	*df*				5			5				
	*F* value				9.19			8.12				
	*p*	ns	ns	ns	<0.01	ns	ns	0.01	ns	ns		ns
Cryptophyceae	*df*	2										1
	*F* value	6.39										9.64
	*p*	0.01	ns	ns	ns	ns	ns	ns	ns	ns		<0.01
Euglenophyceae	*df*			5								
	*F* value			5.46								
	*p*	ns	ns	<0.05	ns	ns	ns	ns	ns	ns		ns
Chlorophyceae	*df*					5	5	5	5	5		
	*F* value					5.3	10.05	4.91	7.45	45.39		
	*p*	ns	ns	ns	ns	<0.05	<0.01	<0.05	0.01	<0.001		ns
Dinophyceae	*df*											
	*F* value											
	*p*	ns	ns	ns	ns	ns	ns	ns	ns	ns		ns
Cyanobacteria	*df*	2										
	*F* value	6.72										
	*p*	0.01	ns	ns	ns	ns	ns	ns	ns	ns	ns	ns

*Note*. DOC = dissolved organic carbon, SRP = soluble reactive phosphorus; ns = not significant (*p* >0.05).

Bacillariophyceae (mainly small *Cyclotella* spp.) represented also an important share of the algal biomass (average 23% of the total grazable biovolume during the study period) and were present in all samples (Figure 4), although in general less abundant than Cryptophycea. The biovolume of diatoms was also significantly different during the study years (*df* = 2, *F* value = 11.22, *p* < 0.01) and highest in 2015, particularly in June represented up to 86% of the total grazable phytoplankton biovolume. Diatom biovolume was significantly affected by nutrients as SRP (*df* = 5, *F* value = 7.32, *p* < 0.01), seston inflow (*df* = 5, *F* value = 8.86, *p* < 0.01), and ice cover (*df* = 5, *F* value = 19.18, *p* < 0.01; Table 1).

Chlorophyceae occurred in 97% of the samples and were particularly abundant during the summer months (Figure 4), notably in August 2013 when a bloom of *Oocystis* spp. represented 75% of the total grazable phytoplankton biomass. The proliferation of green algae coincided, although not significantly correlated, with water temperature in 2013 (*df* = 5, *F* value = 3.6, *p* = 0.07; Table 1). Other green algae were present throughout the entire study (Figure 4), and the most represented groups were small Chlorococcales and *Tetraedron* sp. (present in 39% and 34% of all samples, respectively). Water transparency (*df* = 5, *F* value = 7.54, *p* < 0.01), NO_2_ (*df* = 5, *F* value = 5.39, *p* < 0.05), precipitation, and seston at lake inflow (*df* = 5, *F* value = 4.67, *p* < 0.05, and *df* = 5, *F* value = 4.98, *p* < 0.05, respectively) were significantly related to the biovolume of Chlorophyceae (Table 1). Small Dinophyceae and Chrysophyceae represented only 5–10% of the total grazable phytoplankton biovolume (Figure 4). Among Dinophyceae, *Peridinium* spp. was the most abundant genus and present in 70% of the samples. Among unicellular Chrysophyceae, the loricated genus *Kephyrion* sp. was most ubiquitous and present in 58% of the samples. Chrysophyceae biovolume was significantly related to SRP (*df* = 5, *F* value = 9.33, *p* < 0.01) and lake seston inflow (*df* = 5, *F* value = 8.24, *p* = 0.01; Table 1).

Cyanobacteria biovolume was significantly higher between June and September in 2013 than 2014 and 2015 (*df* = 2, *F* value = 4.21, *p* < 0.05; Figure 4; Table 1) due to the presence of short filaments of *Cyndindrospermum* sp. and colonial *Aphanoteche* sp. representing up to 8% of the grazable phytoplankton biovolume.

### 3.3 Seston Fatty Acids

**Figure 5:**
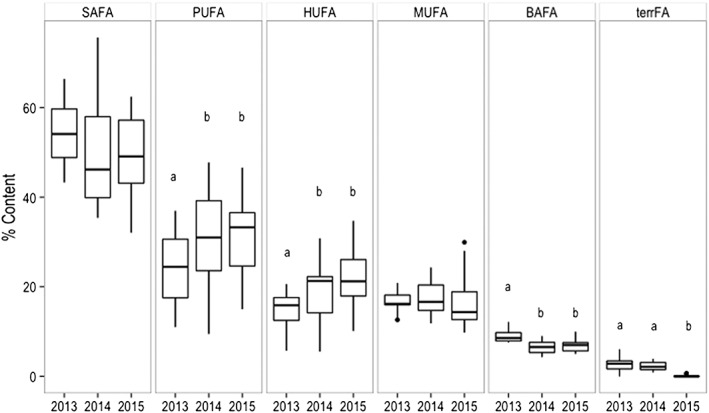
Saturated (SAFA), monounsaturated (MUFA), polyunsaturated (PUFA), highly unsaturated (HUFA), bacterial (BAFA), and, terrestrial fatty acids (terrFA) content (%) in seston (epilimnion and metalimnion) during the study years. The box borders indicate the lower and upper quartiles, the centerline is the mean, and the whiskers extending out from the box indicate the maximum and minimum point outside the upper and lower quartiles. Black dots are outliers. Different letters account for statistical significant differences (ANOVA with Tukey's HSD test *P* < 0.05).

Seston FA (Figure 5) were mainly composed by saturated fatty acids (SAFA) that were higher in 2013 but not significantly different during the study years (average 54.77% ± 1.84, 49.16% ±3.51, and 49.31% ±5.82 in 2013, 2014, and 2015, respectively). The relative contents of PUFA and HUFA were lowest in 2013 (PUFA = 24.79% ± 7.23 and HUFA = 14.84% ± 3.87), higher in 2014 (PUFA = 30.88% ± 9.49 and HUFA = 19.39% ± 5.79), and highest in 2015 (PUFA = 49.31% ± 9.09 and HUFA = 21.48% ± 6.11). In contrast, BAFA were highest in 2013 (8.87 ± 1.22), lower in 2015 (6.74 ± 1.31,) and lowest in 2014 (6.48 ± 1.34). In 2013, HUFA and PUFA were significantly lower compared to 2014 and 2015 (*df* = 2, *F* value = 8 and 3.78, respectively, *p* < 0.05), BAFA were significantly higher in 2013 compared to 2014 and 2015 (*df* = 1, *F* value = 23.45, *p* < 0.001), and TerrFA were significantly lower in 2015 compared to 2013 and 2014 (*df* = 2, *F* value = 42.47, *p* < 0.001).

**Figure 6:**
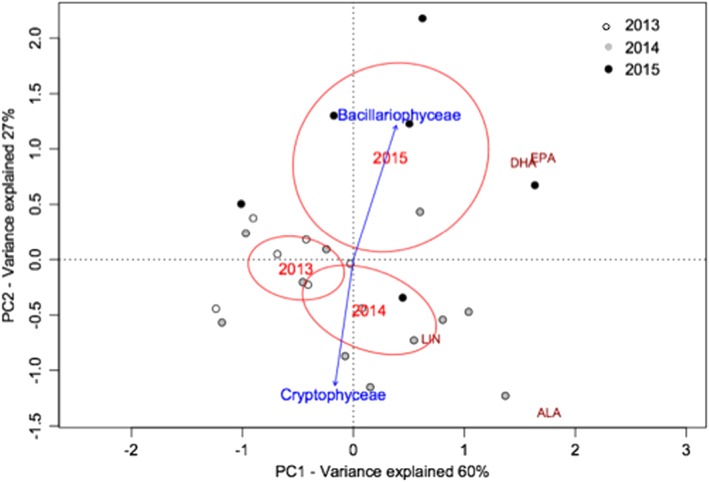
Principal components analysis ordination of polyunsaturated fatty acids in seston of Lake Lunz during the study period at the investigated depths. EPA = eicosapentaenoic acid, DHA = docosahexaenoic acid, LIN = linoleic acid, ALA = α‐linolenic acid. The vectors indicating the phytoplankton taxa associated with PUFA are statically significant (Envfit, *p* < 0.05).

The PUFA ordination (PCA) showed the variation of these FA among phytoplankton taxa (Figure 6). The first PCA axis explained 60% of the variance in the PUFA distribution and clearly separated 2013 from 2014 and 2015 (centroids goodness of fit, *p* = 0.001), that is, the years when Cryptophyceae and Bacillariophyceae, both rich in LIN, ALA, EPA, and DHA, were most abundant. The second PCA axis (PC2) accounted for 27% of the variance and separated the lake samples typical from the winter period, dominated by Cryptophyceae and mostly characterized by LIN and ALA, from the summer community dominated by Bacillariophyceae and mostly associated with EPA and DHA.

**Figure 7:**
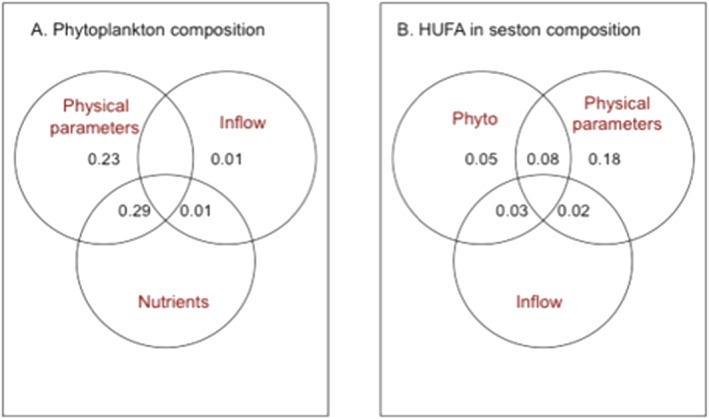
Venn diagram of variation partitioning of the drivers for (a) phytoplankton composition and (b) highly unsaturated fatty acids (HUFA) composition in seston. Lake physical parameters: temperature, conductivity, mixing depth, transparency, and ice cover. Inflow: seston inflow and precipitation. Nutrients: SRP and NO_2_‐N. Phyto = phytoplankton community composition expressed as relative biovolume.

The variation partitioning (Figure 7a) of grazable phytoplankton showed the importance of lake physical parameters (temperature, conductivity, mixing depth, transparency, and ice cover, 23% of explained variance). Nutrients alone could not explain any of the phytoplankton taxonomic composition variance, but in combination with lake physical parameters explained 29% of the variance.

The HUFA composition was mainly determined by lake physical parameters (18% of explained variance), the grazable phytoplankton composition expressed as taxa biovolume (5% of explained variance), and the shared effect of lake physical parameters and seston composition that explained 8% of the variance. The combined effect of seston inflow and precipitation with seston composition explained 3% of the variance, while seston inflow and precipitation together with lake physical parameters explained 2% of the HUFA variance.

## 4 Discussion

### 4.1 Environmental Parameters

The highly irregular duration of full ice cover within these 3 years can be considered an important sentinel for changes in lake thermal stability. By contrast, the nonsignificant increase in surface water temperature indicates that the overall increase of this parameter recorded over the last decades in Lake Lunz (Kainz et al., 2017) cannot be represented within such a short time period. A consequence of the thermal changes in lake water is the increasing temperature difference between the surface layers and hypolimnion. The progressive decrease of hypolimnetic temperature is typical of dimictic lakes, where continued decrease in deep‐water temperatures has been observed since the beginning of this century (Gerten & Adrian, 2002). The ongoing separation between the hypolimnion and surface waters has also been evident from the oxygen depletion at the bottom layer during the last 2 years of this study. The fact that changes in oxygen were not correlated with temperature is likely due to reduced mixing and thus lower exchange of oxygen between shallow and deep water.

Decreasing water transparency has been associated with intense rainfall (Gaiser et al., 2009), which supplies lakes with more particulate and dissolved matter (Jennings et al., 2012; Williamson et al., 2014). The observed correlation between decreasing lake water transparency and increasing conductivity and precipitation was likely due to increased particle loads from stream water inflow from the lake catchment.

### 4.2 Phytoplankton Dynamics

The most grazable size fraction (<30 μm) of the phytoplankton community was characteristic of clear and oligotrophic subalpine lakes (Gallina et al., 2013). The highest biovolume occurred in the metalimnion and was thus associated with the base of the thermocline, which was evident for species with high sinking rates such as diatoms. High phytoplankton biovolume occurred during summer months due to higher temperature and nutrient input (Figure S3), while lower biovolume occurred during winter months and in April–May, associated with low Secchi depth. The main species (typically *Cryptomonas* sp., *Rhodomonas* sp., *Cyclotella* spp., and small Chlorophyceae) reoccurred in all 3 years. Although the presence of these taxa remained rather stable, their biovolume was associated with changes in temperature, seston inflow, and duration of ice cover. These parameters are subject to changing climate conditions (e.g., Hampton et al., 2017) and suggest that ongoing changes in lake temperature, precipitation, and duration of ice cover of Lake Lunz (Kainz et al., 2017) may continue to affect the phytoplankton composition in the future. The significant increase in diatom biovolume with decreasing lake mixing depth and ice cover (Table 1) was related to increased lake water inflow and possibly to increased supply nutrients from catchment runoff (Jeppesen et al., 2009). Considering the dramatically reduced duration of snow and ice cover in Lake Lunz (Kainz et al., 2017), one can also expect reduced release of soil nutrients from snow melt and consequently a related decrease of the diatoms proliferation as what occurred during 2013 and 2014. The interaction of nutrients (SRP and NO_2_) and physicochemical parameters explained the variation in phytoplankton group biovolume, corroborating the hypothesis that phytoplankton growth in oligotrophic ecosystems is sensitive to temperature and increased nutrient supply (Persson et al., 2008; Villaescusa et al., 2016). The significantly increased nitrite concentrations, notably in the epilimnion, may account for nitrogen enrichment due to atmospheric deposition, as currently detected in the northern hemisphere (Bergström & Jansson, 2006), which can promote phytoplankton growth also in oligotrophic lakes (Reichwaldt & Ghadouani, 2012). Indeed, precipitation was a significant parameter determining the Chlorophyceae biovolume, which was also positively correlated to NO_2_ availability (*R* ^2^ = 0.21, *p* < 0.05).

During the first 2 years, the reduction of ice cover may have affected the mixing regime and consequently favored buoyant species such as colonial green algae (e.g., *Oocystis* spp.) and Cyanobacteria (e.g., *Aphanoteche* sp.; Hampton et al., 2014; Huisman et al., 2004). This may account for relatively minor direct effects of temperature change on the phytoplankton community dynamics but more indirect influence of ice cover duration and mixing regime by favoring buoyant species.

### 4.3 FA Composition

The FA composition in seston was significantly different in 2013 compared to the other study years, accounting for a response in nutritional quality to the detected change in the physicochemical lake parameters (e.g., warmer temperature and reduced lake ice cover) and phytoplankton taxonomic composition (e.g., higher biovolume of Chlorophyceae and Cyanobacteria). The typical seasonal phytoplankton composition in Lake Lunz, mostly dominated by Cryptophyceae and Bacillariophyceae, notably during the coldest years 2014 and 2015, supplied highly nutritious food to consumers composed of long‐chain PUFA, including EPA and DHA. This particular PUFA composition in seston was supported by low temperature and SRP availability, as such environmental conditions likely promoted the presence of stenothermic and slow growing cells, such as PUFA‐rich Cryptophyceae and Bacillariophyceae, mostly favored by large rainfall events and water inflow notably during spring months (Figure S2).

In alpine lakes, typically low water temperature and low nutrient supply are favorable for PUFA‐rich algae and clearly differentiate from the nutritional quality of seston from shallow, warm, and nutrient‐rich lakes that often contain algae poor in long‐chain PUFA (e.g., Müller‐Navarra et al., 2004). Nutrient‐rich conditions may result in competitive exclusion of algae rich in long‐chain PUFA (Downing et al., 2001) and lead to rapid growth of few dominant species, mainly characterized by simple life histories and rapid reproduction (Rasconi et al., 2017). Eutrophic lakes exposed to global warming are indeed known to promote high algal biomass but characterized by species typically forming mono‐specific bloom (e.g., Cyanobacteria), generally lacking PUFA (von Elert, 2002). Such species are thus often of poor nutritional quality and can become even toxic, thus inadequate to support consumer development (von Elert, 2002).

The observed switch in the FA composition from high PUFA to high BAFA in 2013 suggests taxonomic changes at the base of the food web from Cryptophyceae and Bacillariophyceae to Chlorophyceae and Cyanobacteria associated to the warmest year (2013) and likely also a different mixing regime. Although detailed information on the heterotrophic contribution to seston FA (i.e., heterotrophic protists; Bec et al., 2010; or fungi; Kagami et al., 2007) is missing, a clear relationship appeared among the phytoplankton taxa and nutritional quality of seston. During 2013, notably the high BAFA content in seston (Figure 5) was likely due to the presence of Cyanobacteria, while in 2014 and 2015, the higher occurrence of Cryptophyceae and Bacillariophyceae contributed to higher nutritional quality and higher PUFA content in seston.

Overall, the strong correspondence between HUFA and physicochemical parameters, which could explain 20% of the related HUFA composition (Figure 7), suggests that the annual occurrence of highly nutritious HUFA for consumers can be sensitive to interseasonal changes in temperature, precipitation, and lake ice‐cover in this subalpine lake. Contrary to what we expected, the phytoplankton composition was not the best predictor for the FA composition. This can be due to some methodological limitations; for example, some colonial algae may have disintegrated and passed through the 30‐μm mesh (such as the highly nutritious Crysophyceae *Uroglena* that were abundant in Lake Lunz during the summer months; unpubl. data), and/or some unidentified organisms, such as planktonic fungi that produce zoospores rich in PUFA (Kagami et al., 2007), may have been present. Alternatively, long‐chain PUFA are essential dietary compounds, especially important during low temperatures, as they become integral parts in cell structures supporting membrane fluidity and cell functioning (Los & Murata, 2004), and their production is directly affected by environmental traits. Algal lipids may be indeed influenced by physicochemical characteristics, which affect photosynthesis such as light intensity, or nutrient availability that may entail biochemical responses on algal metabolism (Guschina & Harwood, 2009). Thus, the particular HUFA composition of seston in Lake Lunz may have been also directly influenced by the recent changes in lake physicochemical characteristics such as precipitation, water inflow, and transparency.

## 5 Conclusions

The phytoplankton taxonomic composition was directly related to the environmental parameters, such as lake ice cover, seston inflow, and nutrient availability determined by irregular runoff from the catchment and climate events. Precipitation was a significant parameter determining the Chlorophyceae biovolume, which was also positively correlated to NO_2_. Moreover, the decreasing period of full ice cover may have affected the mixing regime and consequently favored buoyant species as colonial green algae and Cyanobacteria. As hypothesized, the dietary quality of seston, as assessed by PUFA, was directly related to the variation of lake physicochemical parameters and the phytoplankton taxonomic composition. The phytoplankton taxonomic composition remained fairly stable, but the dominance of the different groups and their relative biovolume differed during this study period. This suggests that the taxonomic composition of phytoplankton in cold and oligotrophic subalpine lakes and their FA composition are likely to respond rapidly to changes in lake physical parameters (such as temperature, transparency, and seston inflow) with potentially long‐term consequences for trophic energy transfer in lake food webs. It becomes clear that more long‐term studies on oligotrophic, cold, and deep lakes are warranted to better understand how dietary quality at the base of the food web responds to warming, changes in nutrient concentrations, and/or weather events. Finally, results from this three‐year study confirmed that as a consequence of rapidly changing physicochemical lake conditions, oligotrophic, subalpine lakes, such as Lake Lunz, may alter their nutritional quality due to reduced growth of PUFA‐rich phytoplankton species such as Cryptophyceae and Bacillariophyceae or due to higher proliferation of PUFA‐poor and nutritionally less adequate species as Chlorophyceae and Cyanobacteria.

## Supporting information



Supporting Information S1
