# Supplementary material for: Seston Fatty Acid Responses to Physicochemical Changes in Subalpine Lake Lunz, Austria
Source: Water Resour Res. 2018 Oct 29;54(10):8442–55. doi: 10.1029/2017WR020959 (PMC6283001; doi:10.1029/2017WR020959)

AGU PUBLICATIONS

Water Resources Research

Supporting Information for

Seston fatty acid responses to physicochemical changes in subalpine Lake Lunz, Austria

S. Rasconi[1], R. Ptacnik[1] and M. J. Kainz[1]

[1]WasserCluster Lunz – Biologische Station, Inter-university Center for Aquatic Ecosystem Research, Dr. Carl Kupelwieser Promenade 5. A-3293 Lunz am See, Austria

Corresponding author: Serena Rasconi (Serena.Rasconi@wcl.ac.at)

Contents of this file

Text S1
Figures S1 to S3

Text S1.

List of the phytoplankton genera with dimensions <30 µm retained for the study and their biovolume assigned using standardized reference data from [*Kremer et al.*, 2014].

**Bacillariophyceae**	Biovolume (log)
Cyclotella catenata	1.698970004
Navicula minuscula	1.656851325
Achnanthes	2.013051105
Nitzschia microcephala	1.97907472
Cymbella turgidula	2.621176282
**Chrysophyceae**	
Kephyrion	1.855790314
Ochromonas	1.153509989
Chrysamoeba	2.614302832
Pedinella	2.48405302
**Euglenophyceae**	
Trachelomonas	3.130378604
**Cryptophyceae**	
Rhodomonas nanoplanktonica	1.635798701
Rhodomonas lacustris	2.203698746
Plagioselmis	2.343469841
**Dinophyceae**	
Peridinium	2.224558959
**Chlorophyceae**	
Chlamydomonas	2.23795518
Tetraedron	2.165320579
Monoraphidium minutum	1.873472879
Selenastrum	1.620134549
Cosmarium pygmaeum	2.31330625
Oocystis	2.115418992
Scenedesmus	2.400498879
Scenedesmus ecornis	2.641597692
Scenedesmus dimorphus	2.608866914
Elakatothrix	2.011333983
Chlorococcales	2.018185667
Ankyra	1.962434509
Keratococcus	2.435228261
Crucigenia quadrata	2.095268066

Cyanobacteria

Aphanothece nidulans	0.747411808
Cylindrospermum	1.75605319
Planktothrix	2.154027359

Figure S1. Number of days of full lake ice cover during the three investigated years.

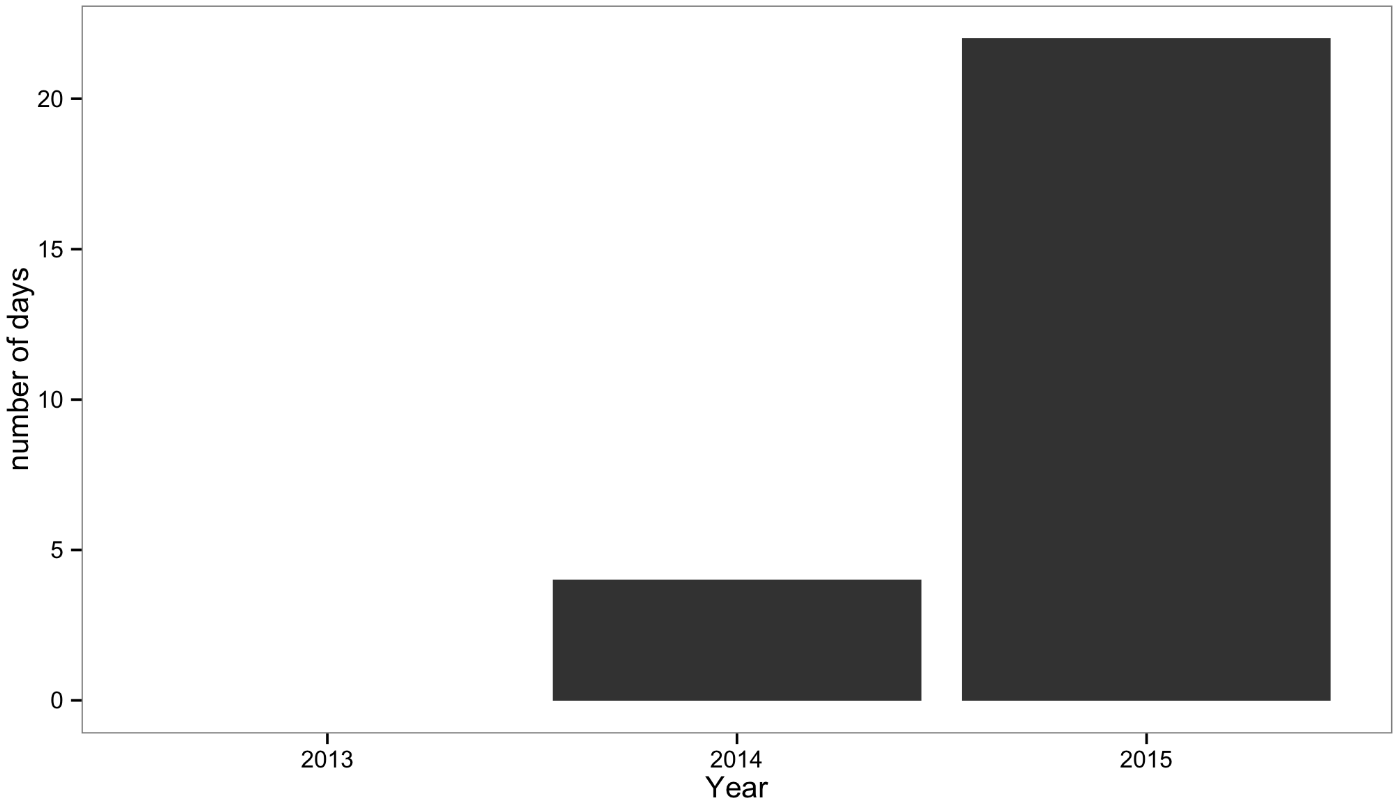

Figure S2. Temporal dynamic during the three years (month.year) of a) precipitation; b) transparency; c) seston inflow.

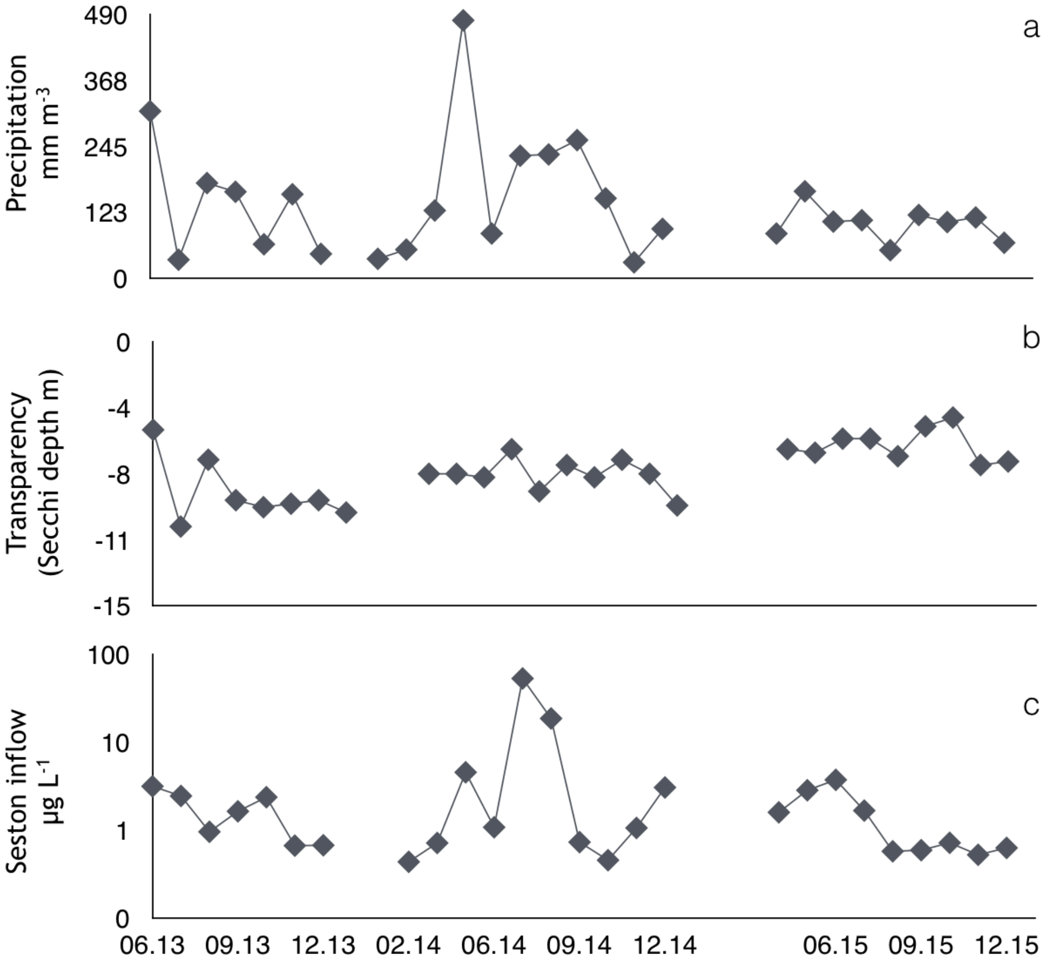

Figure S3. Temporal dynamic at the three investigated depths during the three years (month.year) of all the measured physicochemical parameters. a) conductivity; b) oxygen concentration; c) dissolved organic carbon, d) total phosphorus; e) soluble reactive phosphorus; f) nitrite; g) nitrate; h) ammonium.

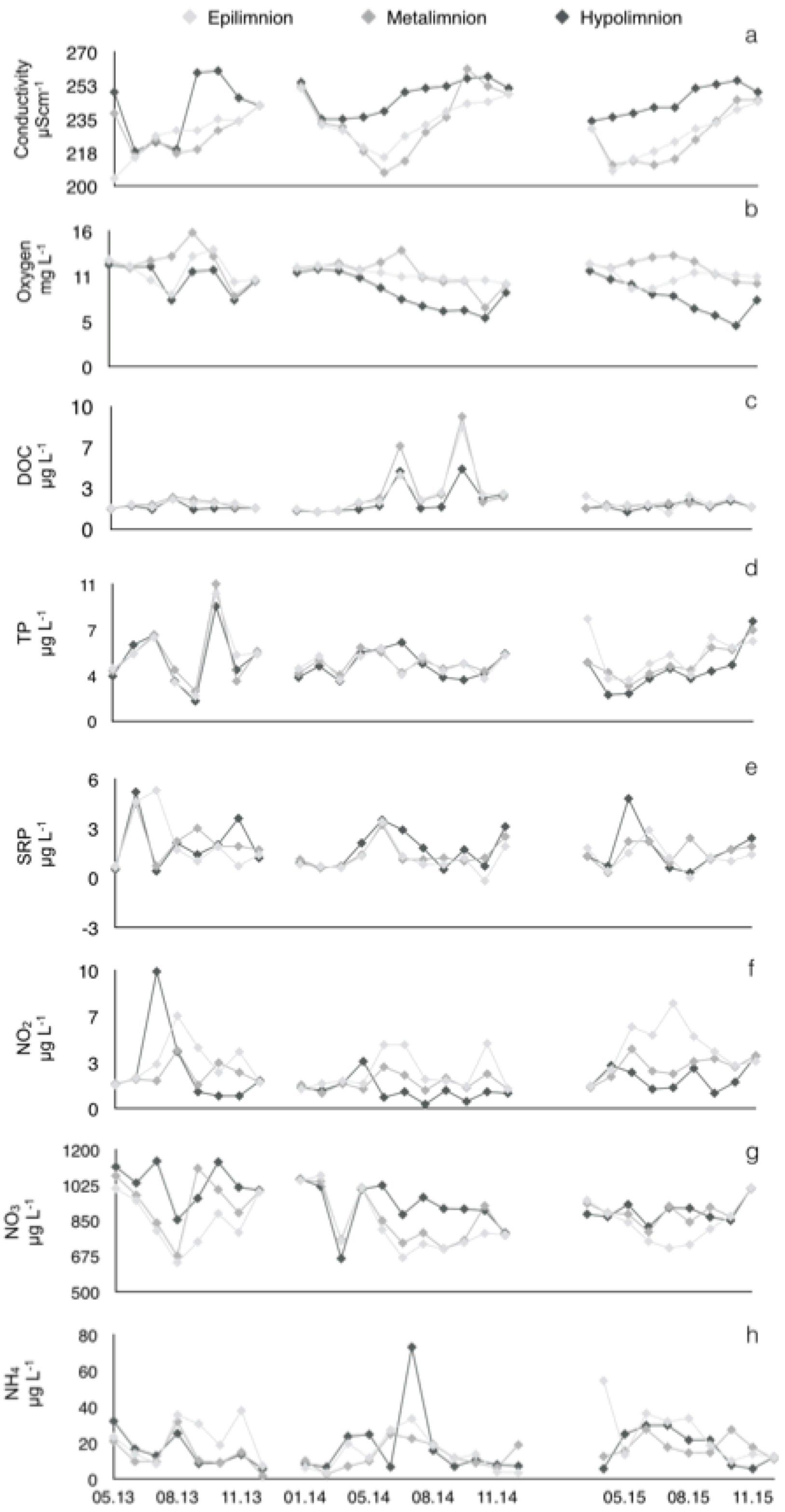

Supplement: Supplementary file 1 — Supporting Information S1 [file WRCR-54-8442-s001.pdf]
